# Evolution of the Illegal Substances Market and Substance Users’ Social Situation and Health during the COVID-19 Pandemic

**DOI:** 10.3390/ijerph18094960

**Published:** 2021-05-07

**Authors:** Jacques Gaume, Elodie Schmutz, Jean-Bernard Daeppen, Frank Zobel

**Affiliations:** 1 Department of Psychiatry, Addiction Medicine, Lausanne University Hospital and University of Lausanne, Rue du Bugnon 23, 1011 Lausanne, Switzerland; elodie.schmutz@chuv.ch (E.S.); Jean-Bernard.Daeppen@chuv.ch (J.-B.D.); 2 Addiction Suisse, Avenue Louis-Ruchonnet 14, 1003 Lausanne, Switzerland; fzobel@addictionsuisse.ch

**Keywords:** substance use, black market, users’ health and social situation, COVID-19, lockdown

## Abstract

The outbreak of the COVID-19 pandemic and the measures taken for tackling it had the potential to lead to deep modifications in the supply of illegal drugs and to impact substance users’ health and social situation. To investigate this, we used mixed methods, i.e., quantitative data collected with a brief questionnaire from substance users receiving opioid agonist treatment in a treatment centre in Switzerland (*N* = 49), and qualitative data obtained using semi-structured phone interviews among a sub-group of participants (*N* = 17). We repeated data collection twice over four weeks to investigate trends over time (*N* = 51 and 14 at wave 2). Findings consistently showed the limited impact of the COVID-19 outbreak on the illegal substance market. Over the two waves, the supply, price and purity of three main illegal substances did not significantly vary. Substance use was estimated as usual by most, trending toward a decrease. The impact of the pandemic on participants’ social situation and health was appraised as low to medium. Nevertheless, a minority of participants reported higher impact and multivariate analyses showed a more important impact for those who were female, younger, and not using multiple substances. This process was implemented quickly and provided an understanding of the short-term impact of the pandemic on drug markets and users.

## 1. Introduction

The outbreak of the COVID-19 pandemic and the measures taken for tackling it had the potential to lead to changes in numerous areas, including the illegal substances market and substance users’ health. At the beginning of the COVID-19 outbreak in Europe (March 2020), the European Monitoring Centre for Drugs and Drug Addiction published a report on the disease’s impact on drug users and drug service providers [1]. For highly vulnerable persons, the risks related to disease include high prevalence of chronic diseases, increased risk of lethal intoxication in cases of infection, the sharing of substance use equipment which might increase infection risk, crowded environments and the disruption of social and health care facilities. The report also highlighted risks related to changes in the illegal substance market, such as disruptions and reductions in the supply of illicit drugs which “could have a range of repercussions especially for dependent drug users and could potentially result in an increased demand for drug services” [1]. At the same time, many countries implemented population lockdowns, reinforced their border controls, and drastically curtailed the transportation of goods and people. This was the case, not only in Switzerland and neighbouring countries, but also in many other countries producing and transporting illegal substances, such as Afghanistan, Iran, Turkey, and the Balkan states for opioids, [2], Colombia, Peru, Bolivia, Brazil and Venezuela for cocaine [3], Morocco and Spain for cannabis [4] and the Netherlands, Belgium, and Czech Republic for various other stimulant substances [3]. This unprecedented situation could have led to deep modifications in the supply and distribution of drugs. There were worst-case scenarios of shortages of substances such as heroin and cocaine, and reduced supplies of cannabis [since part of this market is locally supplied [4], but they were only hypothetical, since there were no observational data nor knowledge of similar situations to predict what course the supply of illegal substances would follow [5].

The impact of the pandemic and the measures taken to address it had not been investigated in detail among substance users themselves. Several publications addressed these issues, but usually discussed only potential effects and/or recommendations, such as ensuring service continuity and accessibility of opioid agonist treatment (OAT) during the pandemic [6,7,8,9,10] without providing original data or direct observations. Since then, research on this topic has been conducted and several studies have been published (see Discussion). Among these, one study showed that COVID-19 patients with substance use disorders had significantly worse outcomes than other COVID-19 patients. Death rates were 9.6% vs. 6.6%, while hospitalizations were 41.0% vs. 30.1%, respectively; this highlighted the need to screen and treat individuals with SUDs as part of the strategy to control the pandemic, along with preventing disparities in access to healthcare support [11].

The present project aimed to overcome this lack of knowledge. In the context of the COVID-19 outbreak, we created a design inspired by rapid assessment processes [12,13], and used a mixed methods model [14], combining both qualitative and quantitative strategies in order to obtain first-hand observations of those who purchase substances in the illegal market. Due to lockdown and social distancing measures, normally open drug scenes were practically deserted and the social and health care facilities for drug users were poorly attended. Therefore, we targeted patients in the opioid agonist treatment (OAT) program at the Addiction Treatment Centre (ATC) of Lausanne University Hospital, because they were still being treated and were in frequent contact with the clinical staff (who knew that many of them were still using illegal substances, such as cocaine, cannabis, or street heroin).

The ATC provides methadone, slow-release oral morphine, buprenorphine, and diacetylmorphine (pharmaceutical heroin) options for these patients, whose treatments are individually tailored. The centre additionally provides support and treatment for social, mental or physical problems, as well as harm reduction information and material (e.g., sterile injection or inhalation kits and condoms, etc.). In order to specifically address the COVID-19 outbreak, the ATC implemented hygiene measures (e.g., social distancing, face masks, hydro-alcoholic solutions and temperature controls), adapted its operating hours to mitigate the flow of patients, decreased visit frequency when appropriate (i.e., certain patients were given several take-home OAT doses) and authorized telehealth services (e.g., consultations by phone or video chats). For the most vulnerable patients, OAT home delivery was introduced. In such cases, some treatments were modified (e.g., oral vs. injected), since hospital monitoring of clinical parameters and emergency care could not be as extensive as it was previously. COVID-19 information, both general and specific to substance users, was provided by clinical staff and through wall posters at the centre.

Our mixed methods research consisted of quantitative data, collected with a brief anonymous questionnaire containing limited information from a large number of patients, and qualitative data, obtained among a sub-group of participants using semi-structured phone interviews containing observations and personal experiences in more depth. In order to investigate trends over time, there were two waves of identical data collection over four weeks. This process could be implemented quickly and provided an understanding of the short-term impact of the pandemic and related lockdown on drug markets and vulnerable drug users.

## 2. Materials and Methods

### 2.1. Quantitative Questionnaire

All ATC patients in OAT during the study period were invited to participate in the quantitative portion (*N* = 79). The clinical staff first used criteria to exclude those who had not bought any illegal substances during the last seven days (*n* = 3), were not able to speak enough French to answer the questionnaire (*n* = 4) or were too intoxicated or mentally unstable (*n* = 2). Eligible patients were then verbally informed of the study purpose and procedures and were reassured that the questionnaire was fully anonymous and would not be seen by any clinical staff. Verbal consent was provided by 49 patients and 21 refused to participate (i.e., 62% participation rate). Participants had access to a quiet zone ensuring confidentiality in completing the questionnaire. A blank envelope was provided, and participants sealed their questionnaire in the envelope and deposited it in a collection box. All procedures were approved by the competent Ethics Committee (*Commission cantonale vaudoise d’éthique de la recherche sur l’être humain*), Project-ID 2020-01015.

Participation was offered to the 70 patients returning to the program during the week of 17–24 April 2020; 49 of them agreed to be interviewed in this first week (wave 1). At this time, Switzerland was under several measures known as “soft-lockdown”, i.e., the closure of all stores and markets (except for food and first necessity goods), schools, restaurants, bars, nightclubs, etc. Gatherings of more than 5 persons were banned. The second wave occurred two weeks later, 4–8 May 2020; this time 51 participants completed the questionnaire. During this period, several lockdown measures were lifted, such as the closure of some stores.

Data were collected using a 2-page, self-reported, paper and pencil questionnaire. The form was developed for this study and designed to be short and clearly written, in order to minimize refusals and difficulties in responding (see original questionnaire in Supplementary Material). The first section focused on the use and purchase of three of the most common illegal substances in Switzerland (heroin, cocaine, and cannabis). Questions on use were 1—‘any use of the substance during the last 7 days (yes/no)’, 2—‘number of days of use’, and 3—‘usual number of episodes of use per day’. Questions on substance purchase were 1—‘where did the substance come from’ and had multiple choices (black market dealer, bought from another user, given by another user, and personal stock), 2—‘amount and purchase price’, separately for typical categories in Switzerland [2,3,4]. These categories included heroin small baggies (0.2–0.5 gr.) and “zip” bags (5 gr.), cocaine small baggies (0.2 gr.) and big baggies (0.8–1 gr.), herbal cannabis (marijuana, weed) and cannabis resin (hashish). For each category, we asked the amount (in grams) purchased and the price in Swiss francs (CHF; CHF 1 ≈ USD 1 ≈ EUR 0.95 at the time of the study). The price per gram for each category was computed by dividing the price per amount purchased. Participants were asked to estimate the purity of the substance on a 4-point scale (‘low’, ‘medium’, ‘high’, and ‘very high’). Additional parameters were provided to help estimate purity, based on standard purity measured in Switzerland [2,3,4]: for heroin 0–10% (low), 11–20% (medium), 21–30% (high), and >30% (very high); for cocaine 0–25%, 26–50%, 51–75%, and >75%; and for cannabis THC content 0–5%, 6–10%, 11–15%, and >15%.

The second part of the questionnaire evaluated the impact of the pandemic felt by participants. Question 1 evaluated the impact on the use of six substances: heroin, cocaine, cannabis, alcohol, prescription drugs (e.g., benzodiazepines), and ‘other drugs’ (any other drugs, none specified). The impact was measured on a 5-point Likert scale with anchors at 1 ‘decreased’, 3 ‘usual’, and 5 ‘increased’. Question 2 evaluated the impact on social and health conditions: 1—social and financial situation, 2—fear of police controls, 3—stealing or racketeering of substances, 4—stress and anxiety, 5—mental health in general, and 6—physical health in general. A 5-point Likert scale had anchors at 1 ‘no impact’, and 5 ‘a big impact’. We also assessed gender, age, and professional status (employed vs. not).

Statistical analyses were mainly descriptive. Categorical variables used N and percentages and continuous variables used medians and interquartile range (IQR, i.e., 25th–75th percentiles), since they did not follow a normal distribution. We compared trends between waves using non-parametric tests (Pearson’s Chi square for categorical variables and Wilcoxon rank-sum for continuous variables). Next, we computed Spearman’s rank correlations between the impact on different substances to see whether individuals would follow similar trends for all substances (i.e., increased or decreased all substances) or show transfer from one substance to another (e.g., decrease heroin and increase alcohol use). Multivariate models then investigated the effect of age, gender, and number of substances used (as a proxy of polysubstance use) on the impact of the pandemic. The effect of professional status was not determined due to the low proportion of employment (e.g., less than 5% were working at wave 2). For each impact measure, a generalized estimating equation (GEE) model was built, taking into account the clustered nature of data (by waves). Age, gender and number of substances were entered into the model simultaneously. We specified an independent correlation structure, with robust standard errors adjusting for waves. Finally, we used Spearman’s correlations to investigate the link between the impact on substance use and the impact on social situation and health. Analyses were conducted using Stata IC 16.0 (StataCorp LLC, College Station, TX, USA).

### 2.2. Qualitative Interviews

The qualitative part of the study included a sub-sample of the participants from the quantitative part. After completing the questionnaire (see above), the clinical staff asked participants whether they were interested in participating in a more in-depth interview on the impact of the pandemic on the black market and the situation of users. After giving them the necessary information about the study goals and procedures, those who agreed to participate were invited to provide a pseudonym and a phone number. This information was given to the research team, who had no access to any other information about the patients.

Due to pandemic safety measures, interviews were conducted over the phone by one of the members of the research team (J.G., E.S., or F.Z.); they were semi-structured and based on an interview guide comprised of four main topics: (1) recent use (substances used and use patterns) and purchase of substances (price, quantity and quality); (2) observed changes in the market in recent weeks; (3) impact of the pandemic on the user’s situation and measures adopted to combat it; and (4) measures that could help users in the current context. No audio recordings were made because of technical and confidentiality reasons (i.e., to avoid any record of illegal behaviours). The analytical material consisted of detailed notes taken during the interviews. As compensation for their time, participants were offered a gift card of CHF 20 value (i.e., USD ~20, or EUR 19) from a large chain of supermarkets and department stores.

A first wave of interviews was conducted 21–29 April 2020. From those who had answered the quantitative questionnaire, 23 (47%) consented to participate and provided contact information. Six of them could not be reached after 5 attempts; thus 17 semi-structured interviews were conducted. The average duration was approximately 30 min, ranging from 15–79 min. A second wave of interviews was conducted three weeks later. The aim was to investigate trends in the participants’ situation, so the interview guide was not changed. One participant was not contacted again due to language difficulties during the first interview, and two others could not be reached after 5 attempts, leaving 14 persons (82%) in the second wave. These interviews took place 12–29 May 2020, three to five weeks (mean 3.7) after the first interview. The average duration was again approximately 30 min, ranging from 12–94 min.

Conventional content analysis was used to derive thematic categories from the interview notes [15]. They were derived and classified by one researcher (E.S. or F.Z.), and then compared across interviews. Data collection was stopped when data saturation was achieved [16]. The derived thematic categories were then synthetized and reviewed by all three researchers who conducted interviews.

## 3. Results

### 3.1. Quantitative Questionnaire

As presented in Table 1, the median age was around 40 years, approximately one fourth of the sample were women, and only about 7% were employed. The socio-demographic variables were statistically comparable across the two waves, with no significant differences.

Substance use. About 43% of the sample used heroin over the last week at wave 1. This proportion was somewhat higher at wave 2 (57%), but this trend was not statistically significant (Χ^2^(1) = 1.96, *p* = 0.16). Around half of the participants used cocaine across both waves. Around half of the participants used cannabis at wave 1, but only a third used it at wave 2 (Χ^2^(1) = 2.52, *p* = 0.11). Looking at combinations of the three substances, about a third of the participants used only one substance, about a third used two, and 10% used all three (about 18% used none, this might be because they used other substances or were included by error). The most common combination at wave 1 was cocaine and cannabis (18%) and the most common at wave 2 was cocaine and heroin (22%). The frequency of heroin use was relatively dispersed at wave 1 (median = 3.5, IQR 2–6) and was lower and less dispersed at wave 2 (median = 2, IQR 1–3, Z = 1.63, *p* = 0.10). Since there were more heroin users at wave 2 (27 vs. 20 at wave 1), this might suggest that there were more users using less often, i.e., more occasional users. The frequency of cocaine use was rather stable, with a median of 3 days at both waves. Cannabis use was relatively stable at a very high median (7 days a week at both waves) but had a larger IQR at wave 1 (1–7 vs. 6–7), indicating fewer occasional users at wave 2. Use episodes per day were also relatively stable across both waves (about 2 and 3 episodes per day for heroin, cocaine, and cannabis).

Substance purchases. There were no major changes in substance purchases. Cocaine was predominantly bought from street dealers (about 2/3 of the sample had bought from the black market), followed by purchases from other users and gifts from other users (about 1/4 each). Heroin and cannabis sources were more diverse. There were no significant differences between waves. Nevertheless, it is worth noting that personal heroin stashes were used by 20% at wave 1, but only by 10% at wave 2. This might coincide with clinical recommendations to build a personal stock of primary substance and preparation materials, which were distributed in the beginning of the pandemic, to avoid widespread withdrawals and related consequences. Substance prices were also relatively stable. Heroin cost about 75 CHF/gr. in small baggies and about 20 CHF/gr. in larger “zip” bags. Cocaine cost about 100 CHF/gr. for small and big baggies alike. Herbal cannabis and resin were around 10 CHF/gr. and did not statistically change across waves, despite a small non-significant decrease for herbal cannabis at wave 2 (median = 8 (IQR 3–10) vs. 10 (6–10) at wave 1). Estimated purity was rather stable at low to medium levels for cocaine and heroin (all IQRs 1–2) and there was a non-significant trend toward lower purity at wave 2 (median = 1). For cannabis, estimated THC levels were higher and did not significantly vary over time.

Impact on substance use. Scales evaluating the impact of lockdown on substance use mainly showed no impact (most medians were at 3, i.e., usual consumption), with a trend toward decreasing use (most IQRs 2–3 or 1–3). This decrease was somewhat stronger at wave 2 for heroin and cocaine (median = 2, IQR 1–3, vs. median = 3, IQR 2–3 at wave 1; Z = 1.51, *p* = 0.13 and Z = 1.40, *p* = 0.16). The use of ‘other drugs’ also decreased more strongly at wave 2 (median = 1, Z = 1.93, *p* = 0.05). Significant correlations of impact with different substances were all positive (see Table 2), indicating that persons increasing one substance would also increase the other; inversely, those decreasing one would decrease the other. Correlations which were significant at wave 1 had similar patterns at wave 2. However, it should be noted that some additional significant correlations were observed at wave 2 only, notably those involving prescription and ‘other drugs’. This might suggest that transfer from one substance to another was minimal at wave 2 but might have happened at wave 1 between prescription and ‘other drugs’ on the one hand, and heroin, cannabis, and alcohol on the other hand, thus cancelling the correlations for these variables at wave 1. Finally, multivariate models investigating the effect of age, gender and number of substances used showed interesting trends. The number of substances used had a significant effect on heroin (B *=* −0.45, SE *=* 0.07, *p* < 0.001), and ‘other drugs’ (B *=* −0.71, SE *=* 0.07, *p* < 0.001); for both substances, a greater number of substances was related to decreased use. A gender effect was also observed for alcohol (B *=* −0.40, SE *=* 0.15, *p =* 0.007) and cocaine use (B *=* −0.63, SE *=* 0.20, *p =* 0.002), with women showing larger decreases. There was also a small effect of age on the impact of the pandemic on use of ‘other drugs’ (which decreased with age, B *=* −0.05, SE *=* 0.003, *p* < 0.001).

Impact on social situation and health. Questions targeting the impact of the pandemic on social situation and health showed little impact overall. The influence of the pandemic on fear of police controls, and stealing/racketeering of substances was particularly low (all medians = 1). The influence on social and financial situations was more important, particularly at wave 1 (median = 3, vs. 1 at wave 2, Z = 1.30, *p* = 0.20). This non-significant trend for the impact to decrease over waves was also observed for stress and anxiety, and physical health in general. Multivariate analyses showed that gender, age, and the number of substances used had significant effects on these measures. A gender effect was observed for the fear of police control (B = 0.59, SE = 0.01, *p* < 0.001), stealing/racketeering of substances (B = 0.51, SE = 0.06, *p* < 0.001), stress and anxiety (B = 0.61, SE = 0.003, *p* < 0.001), and mental health in general (B = 0.39, SE = 0.07, *p* < 0.001); for all four dimensions, women experienced a stronger impact of the pandemic than men did. There was also a small effect of age impacting on the fear of police control (B = −0.02, SE = 0.01, *p* = 0.047) and stress and anxiety (B = −0.01, SE = 0.001, *p* < 0.001) which decreased with age; while the effect on mental health in general increased with age (B = 0.02, SE = 0.01, *p* = 0.006). The number of substances used was related to decreases in experienced impact on stress and anxiety (B = −0.14, SE = 0.06, *p* = 0.01) and physical health in general (B = −0.24, SE = 0.09, *p* = 0.007), while the fear of police control increased (B = 0.11, SE = 0.02, *p* < 0.001).

Finally, we computed correlations between influence of the pandemic on substance use and the influence on social situation and health (Table 3). At wave 1, a greater impact on social and financial situation was related to decreases in cocaine and cannabis use. Conversely, a greater impact on mental health was related to increases in heroin use. These correlations were not significant at wave 2. There was another pattern of correlations showing that stress and anxiety, mental health, and physical health in general were related to increases in prescription drug use (at both waves). All three health dimensions, as well as social and financial situation, were related to increases in alcohol use, but only at wave 2.

### 3.2. Qualitative Interviews

The first wave of interview respondents comprised 13 men and four women. The age range for those who answered was 18–59. The second wave sample contained nine men and four women.

Trends in substance use. A small majority of participants experienced changes in substance use in the context of the pandemic. About half of all users appeared to maintain their use levels, while about one quarter seemed to reduce it. Some participants increased their use, or experienced a decrease followed by an increase. Those who decreased their consumption explained it by naming the following factors: worse quality of substances; fewer external solicitations, such as invitations from friends to get together and use; less sharing of substances by friends and fellow users; and having prior willingness to reduce consumption. Users who increased their consumption blamed it on the boredom caused by the pandemic health recommendations to stay home and limit social interactions.

Substance purchases. Most participants who bought heroin noticed very few changes in this market. There were no major shortages, and price and quality were considered stable compared to the prior situation. Anecdotally, one participant indicated that the only difference he observed was that the dealer wore a hygiene mask. However, one participant among six who bought “zip” bags (~5 gr) mentioned that it was harder to find a dealer. Another participant said he had stopped using heroin (in the beginning of the pandemic) since the quality was too bad. One participant (who did not use heroin) mentioned that she heard that fellow users had gathered their purchase and went to Geneva to avoid bad quality products in Lausanne.

Regarding cocaine, most participants bought small “baggies” (0.2 gr.) from street dealers in Lausanne, where prices were relatively stable (CHF 15–20 for 0.15–0.20 gr.) and not different from pre-lockdown prices. Some mentioned that it was very easy to find cocaine, and two even noted that dealers were more and more enterprising, while only one said it was harder to find street dealers. A few also mentioned that there was an increase in scams; two persons mentioned an increase in prices (CHF 20 vs. CHF 15 for small baggies), one complained about very bad quality in two purchases, and one was thinking that dealers either “cut” more or decreased the quantity for the same price.

The cannabis market was reported as being the most affected by the lockdown situation, especially for resin (hashish). Shortages seemed to arise, along with price increases and scams. One participant mentioned that resin was very bad and made him throw up. There were also mentions of scams related to herbal cannabis; one person mentioned it contained mostly CBD (i.e., cannabis with high levels of cannabidiol but less than 1% THC, which is legally sold at a lower price in Switzerland). Participants mentioned prices between CHF 12 and 18 per gram, which is relatively high. Resin, in particular, had very high prices.

Influence on health and social situation. Participants mostly mentioned issues related to mental health and lack of social interactions. The majority did not mention such issues in the first interview, but about half of them did in the second wave. In the first wave, the main mental health issues were anxiety, depression, boredom, and loneliness. The causes of anxiety were usually not pinpointed but seemed related to states of constant tension. Some said that a sense of panic was perceptible among users in the beginning of the pandemic, in fear of impending shortages and downfalls in the quality of substances. Depression, boredom and loneliness were mostly related to the necessity to stay home and have fewer direct social contacts. In the second wave, participants mentioned a lack of direct social ties and anxiety. A lack of social ties included loneliness, not being allowed to meet with parents or family members who live or work in nursing homes, and a lack of tactile contacts (e.g., not even being able to shake hands to greet others). Anxiety was related to fear of facing a possible second COVID-19 pandemic and mandatory vaccination. A few even talked about conspiracy theories, which they personally believed in or were hearing among friends and fellow users.

An important majority experienced no impact of the pandemic on their physical health. In the first wave, only two of them felt an increase in chronic pain, and a third had a COVID-19 infection with medium to severe symptoms. By the second wave, two of these could not be reached, and the third’s condition had not changed (i.e., still having chronic pain related to change in OAT). One more reported recent gastrointestinal discomfort due to decreased physical activity and increased food intake.

Finally, some participants spontaneously mentioned discomfort related to police activities. There was increased police presence, particularly around Riponne Square, which is known as the meeting point of substance users and outsiders in Lausanne; it was seen as the enforcement of social distancing by most, but several said that the police were also more active than usual on stopping drug deals and use. Most participants thought that this did not impact their substance purchases and use in the end but did add a threat to their current situation.

Suggested measures to help substance users in the pandemic context. Surprisingly, most participants had few or no suggestions. During this segment of the interview, they often approved of the measures already taken, and often mentioned following COVID-19 health recommendations (e.g., seldom leaving their house, frequently washing hands, and respecting social distancing). In the second wave of interviews, more than half of them were still maintaining their health precautions.

Nevertheless, several suggestions were received, mainly during the first wave. A few participants noted a need for extending the opening hours of the city’s supervised drug consumption room (DCR). Such a facility, where substances can be injected or inhaled safely (under supervision) is available in Lausanne but has restricted hours (11.00 AM to 7.30 PM). Suggestions were to open the facility earlier in the mornings to avoid outdoor substance use, since most public toilets were closed for pandemic control, or even to open another DCR at the ATC. A single person wanted extended schedules, because they required greater flexibility in time slots to come and receive treatment at the ATC. Additional suggestions concerned communications regarding the pandemic and related risk reduction. Some participants would have appreciated health care providers providing substance users with more reliable information about the pandemic, in order to counteract conspiracy theories or contradictory information. Others would have appreciated health care providers discussing risks related to the quality of substances, so they could avoid the use of low-quality products.

## 4. Discussion

The findings of this mixed methods project consistently showed the limited impact of the COVID-19 outbreak on the illegal substance market and substance use among the studied sample. Despite the unprecedented barriers to the transport and supply of illegal substances that were created to contain the pandemic, both quantitative and qualitative data showed minor differences in the market. Over the two waves of quantitative data collection covering periods with various levels of lockdown measures, the use, price and purity of three main illegal substances did not significantly vary. Substance prices and purity basically mirrored those measured in studies before the pandemic [2,3,4]. Nonetheless, there was some variability and non-significant trends indicating small effects that our limited study design may not have captured. Data from the qualitative study similarly indicated relatively stable conditions in the drugs black market. There seemed to be no shortages and few changes in heroin purchases. It also appeared that the cocaine market remained relatively stable, although the price of “small baggies” rose slightly and there were periods with more proactive dealers in the streets. However, the cannabis market was a different case, particularly for resin (hashish). There were several indications of restricted supplies, together with increases in price and scams. One participant mentioned very bad resin making him vomit, which might indicate the production of low-quality resin adulterated with synthetic cannabinoids. This had not been seen in western Switzerland, but recently appeared in Bern and Zurich and was connected to severe intoxication and deaths in Europe [17]. Other explorations of the illegal substance market during the COVID-19 pandemic outbreak in Switzerland showed similar results [18]. Indeed, while triangulating analyses of wastewater, used syringes, and drug seizures, interviews with the heads of five regional drug squads, and analyses of purchases on *darknet* sites selling drugs, we showed a rather stable situation and limited impact of the pandemic on substance supply, prices, and quality, except for a cannabis resin shortage [18].

Recent reports from the European Monitoring Centre for Drugs and Drug Addiction (EMCDDA) and the European Union Agency for Law Enforcement Cooperation (Europol), and from the United Nations Office on Drugs and Crime (UNODC) have shown heterogeneous situations at both the country and substances levels [19,20]. While some countries had temporary shortages, price increases, and decreases in purity for some substances, others, such as Switzerland, had more stable situations. Shortages of cannabis resin were also seen in other European countries [20,21]. Despite this, analyses of specialized *darknet* platforms showed a significant increase in cannabis purchase during the COVID-19 outbreak [18], a trend also found in the United Kingdom, Germany, the Netherlands, and France [22].

At this stage of the pandemic, it seemed that importation and supply networks were little affected and continued to operate as usual in Switzerland (with some occasional latency, which eventually may have affected the quality and price). It also appeared that the market of substances imported from far countries such as Afghanistan (for heroin) and Latin America (for cocaine) was less impacted than was the market of substances imported from near countries such as Morocco (hashish) and Spain (herbal cannabis), or locally produced (herbal cannabis). The pandemic in Spain, which was among the most serious in Europe at the time, may explain these unexpected findings. It might also be that the cocaine and heroin markets are strongly established and sufficiently well-organized enough to overcome law enforcement obstacles such as reinforced border controls, drastic decreases in goods and people transportation, population (consumer) lockdowns, social distancing measures and other controls. Organized crime groups remained resilient and adapted their modi operandi to the current situation, further exploiting secure communication channels and adapting transportation models, trafficking routes and concealment methods [20].

Questions investigating the impact of the pandemic on participants’ substance use also indicated minor impact overall. The quantitative and the qualitative data were in the same direction. Substance use was estimated as usual by most, trending toward a decrease. Similar findings were seen in online surveys among substance users in Switzerland [23], Europe [24], and Canada [25], or in wastewater analyses in European cities [26]. In those countries, decreases were more frequent for stimulants (e.g., cocaine, MDMA), mainly due to the closure of the night scene. Additionally, consistent with these findings, a survey of more than 36,000 adult substance users in Europe [27] suggested that the use of alcohol, tobacco, cannabis and other illicit substances remained unchanged for around half of the respondents during the first wave of the pandemic. Among those who changed, overall patterns suggested that more users tended to reduce rather than increase their alcohol use, whilst the opposite was observed for tobacco use and for cannabis use; there was no clear pattern of change for illicit drug use [27].

Analyses of correlations of the impact of the pandemic with the different substances indicated that users generally decreased or increased all substances and did not transfer from one substance to another. However, correlations of prescription drugs and ‘other’ drugs with heroin, cannabis, and alcohol were significant at wave 2, but not at wave 1, suggesting that some transfers from one of these drugs to another might have cancelled the correlations found at wave 1. In a study among thirty subjects with substance use disorder (not receiving OAT), hair analysis showed that samples positive for heroin, cocaine, MDMA and cannabis fell considerably during the lockdown while the consumption of benzodiazepines and alcohol followed the opposite trend [28]. Some were concerned that shortages in heroin supply would lead to the consumption of other substances, such as fentanyl and its derivatives, or resold pharmaceutical products, such as benzodiazepines and buprenorphine [19]. It seems that this was not the case in Switzerland, or at least that it was only temporarily scarce. Similar patterns were also seen in the Czech Republic [29].

The impact of the pandemic on participants’ social situation and health was appraised as low to medium in both the qualitative and the quantitative data. There were no significant differences across the two quantitative waves, despite a slight trend toward decreased impact. However, there was relatively high variability in the scales that was captured in semi-directive interviews as well. A minority of participants indeed reported higher impact related to anxiety, boredom, depression and lack of social contacts. Multivariate analyses also nuanced overall findings and showed that the impact was more important for those who were female, younger, and using a low number of substances, and less important for those who were male, older, and using multiple substances. A recent study showed similar patterns in France, where the impact on substance users’ health and social situation was heterogeneous and depended on substance use patterns and socio-demographics. A particular burden on mental health was also noticed there [21]. In a study among outpatients and residential inpatients suffering from substance use disorders and/or behavioural addictions recruited across Italy [30], the impact of the COVID-19 pandemic was shown to be related with high rates of psychopathological symptoms. Nevertheless, psychopathological burden was globally higher among residential patients than among outpatients.

Correlations of the influence of the pandemic on substance use with social situation and health showed contrasting findings at wave 1, with a higher impact on social and financial situations related to decreases in cocaine and cannabis use, and higher impact on mental health related to increases in heroin use. These findings might indicate that the social and financial situation during wave 1 (with harder lockdown measures) might have had a stronger impact on cocaine and cannabis use. This could be due to fewer social interactions and occasions to use. Similar findings were observed in Europe [24] and among recreational users in Switzerland [23]. It could also be related to lower financial resources to invest in substances, as seen in France [21]. Conversely, the positive correlation of heroin use with mental health impact might reflect the use of heroin to cope with mental health issues triggered by the pandemic and lockdown measures. Coping might also explain the significant correlations of stress/anxiety, mental health, and physical health in general with increases in prescription drugs and alcohol use, and the correlation between a higher impact on social situation and increase in alcohol use at wave 2. Several studies pointed to increased use of heroin, alcohol, and prescription drugs, such as benzodiazepine, related to mental health issues and coping behaviours [24,31,32,33].

The last point addressed in our study concerns the measures and adaptations taken by health care and social institutions and potential suggestions from participants. Qualitative findings indicated that the measures taken and adaptations were globally appreciated and valorised by participants. Hygiene measures and health recommendations, such as staying at home, frequently washing hands, and respecting social distancing seemed to be accepted and followed by most participants. Among the suggestions provided, most wanted additional information and more drug consumption rooms. In one online survey on the impact of COVID-19 on youth mental health, substance use, and well-being in Canada, respondents requested that high-quality information about COVID-19, mental health and substance use supports be made available to help them [25].

Adaptations made at the ATC included extending opening hours to regulate patient flows, decreasing the frequency of individual visits by allowing take-home and home delivery of OAT doses and telehealth services. We recorded only one criticism related to a change in treatment route; this patient used to receive diacetylmorphine by injection but had to switch to oral diacetylmorphine since he received treatment at home. Injections are not allowed outside the hospital due to lack of monitoring of clinical parameters and emergency care. All other comments were favourable toward the adaptations implemented. Beyond the mandatory changes required by the urgency of the COVID-19 crisis, one ponders which of those measures might be sustained in the long run. Similar measures were implemented elsewhere [34,35]. Additional measures were also proposed, such as improving addiction treatment access using telehealth encounters for OAT induction [36,37], substance use decriminalization [38], and safe supply of opioid [39] and other substances, including stimulants [40]. Further research should investigate their potential impact on substance use behaviours, substance use disorder outcomes, treatment retention, intoxications, and mortality, as well as long-term physical, mental, and social outcomes.

In the context of the outbreak of COVID-19, we opted for a design inspired in rapid assessment processes [12,13] and combined quantitative and qualitative methods to collect first-hand observations by substance users purchasing drugs in the illegal market. This methodology quickly furnished valuable findings directly reported to the clinical staff and other local and national institutions [41,42]. Nevertheless, it was accompanied also by several scientific limitations. First, we used anonymized questionnaires in the quantitative phase of the study in order to avoid patients refusing to provide written records of sensitive illegal activities. As a result, we were unable to conduct follow-ups assessing the evolution of individual situations over time via more advanced longitudinal statistical methods. This limit was partly offset with a qualitative follow-up of a sub-group of participants. Additionally, the high participation rates overall, along with the high follow-up rate in the qualitative phase lend strength to the research. Then, we used an ad hoc questionnaire, developed for this project. This was not validated, and no reliability tests were performed. One limitation of the qualitative part of the study was the absence of audio recording and verbatim transcription. Our thematic analysis relied on interview notes, which might have introduced bias. Additionally, it should be noted that our findings are limited by our sample, which was comprised of patients receiving OAT in the Addiction Treatment Centre of a University Hospital in Switzerland. Our country is among the most wealthy in the OECD and has a high performing health system [43], including a universal mandatory health insurance system with virtually 100% coverage [44]. OAT is included in this coverage, and most patients also receive social welfare support. The participants in this study may have higher social integration, more frequent clinical follow-up, and have lower risk of opioid withdrawal or other substance-related consequences, than do others using substances but not receiving OAT. Recent studies suggest that those who are more vulnerable, such as the homeless [45] and those who have lower socioeconomic status [46,47], are more affected by the pandemic. Further empirical research should address the plight of these populations. Finally, our analysis focused only on the first wave of the COVID-19 outbreak. Future research should investigate the impact of subsequent epidemic waves and the long-term effects of the crisis on the illegal substance market and on the health and social situation of drug users.

## 5. Conclusions

This mixed-methods study indicated the limited impact of the COVID-19 outbreak on the illegal substance market. Despite the unprecedented measures taken such as reinforced border controls, drastic decreases in goods and people transportation, population lockdowns, social distancing measures and other controls, the supply, price and purity of the three main illegal substances in Switzerland did not significantly vary over the two waves of data collection. The impact of the pandemic on participants’ social situation and health was appraised as low to medium. Nonetheless, a minority of participants reported a higher impact related to mental health and a lack of social contacts and multivariate analyses showed that the impact was more important for those who were female, younger, and not using multiple substances. The present project aimed to rapidly overcome the lack of knowledge about the evolution of the illegal substances market and substance users’ social situation and health during a major pandemic crisis. Several publications addressed this issue, but usually discussed only potential effects and/or recommendations and did not provide observational data. In spite of the limitations related to our sample and methods, we were able to show that there were no deep modifications in the supply of illegal drugs and no substantial impact on substance users’ health and social situation. Nevertheless, further research should address the plight of more vulnerable populations, as well as the long-term effects of this crisis.

## Figures and Tables

**Table 1:** Descriptive statistics for questionnaire measures.

	Wave 1 (*N* = 49)	Wave 2 (*N* = 51)
	*N* (%) or Median (IQR)	*N* (%) or Median (IQR)
Sociodemographic variables		
Gender, women (vs. men)	11 (22.9%)	12 (27.3%)
Age	39 (32–50)	41 (34–48)
Professional status, employed (vs. unemployed)	4 (8.7%)	2 (4.9%)
Substance use over the last week (used vs. not used)		
Heroin	21 (42.9%)	29 (56.9%)
Cocaine	25 (51.0%)	25 (49.0%)
Cannabis	25 (51.0%)	18 (35.3%)
Combination of substances		
Heroin only	5 (10.2%)	8 (15.7%)
Cocaine only	4 (8.2%)	5 (9.8%)
Cannabis only	6 (12.2%)	4 (7.8%)
Heroin and cocaine	6 (12.2%)	11 (21.6%)
Heroin and cannabis	4 (8.2%)	5 (9.8%)
Cocaine and cannabis	9 (18.4%)	4 (7.8%)
Heroin, cocaine, and cannabis	6 (12.2%)	5 (9.8%)
None of the three	9 (18.4%)	9 (17.6%)
Number of substances used (0–3)	2 (1–2)	1 (1–2)
Frequency of use (days per week over last week)		
Heroin	4 (2–6)	2 (1–3)
Cocaine	3 (1–5)	3 (2–5)
Cannabis	7 (1–7)	7 (6–7)
Quantity (usual number of episodes of use per days)		
Heroin	2 (2–4)	2 (1–3)
Cocaine	2 (2–3)	3 (1–5)
Cannabis	3 (2–5)	3 (2–5)
Where did heroin come from (multiple answers possible)		
Personal stash	4 (19.0%)	3 (10.3%)
Given by another user	4 (19.0%)	8 (27.6%)
Bought from another user	10 (47.6%)	11 (37.9%)
Black market dealer	7 (33.3%)	10 (34.5%)
Where did cocaine come from (multiple answers possible)		
Personal stash	1 (4.0%)	1 (4.0%)
Given by another user	5 (20.0%)	1 (4.0%)
Bought from another user	6 (24.0%)	7 (28.0%)
Black market dealer	15 (60.0%)	17 (68.0%)
Where did cannabis come from (multiple answers possible)		
Personal stash	4 (16.0%)	4 (22.2%)
Given by another user	9 (36.0%)	7 (38.9%)
Bought from another user	12 (48.0%)	7 (38.9%)
Black market dealer	4 (16.0%)	3 (16.7%)
Price (CHF per gram)		
Heroin small baggies (typically 0.2-0.5 gr.)	75 (71–100)	75 (75–100)
Heroin “zip” bag (typically 5 gr.)	20 (20–22)	22 (20–26)
Cocaine small baggies (typically 0.2 gr.)	100 (75–133)	100 (75–111)
Cocaine big baggies (typically 0.8-1 gr.)	100 (89–100)	100 (100–100)
Herbal cannabis (marijuana, weed)	10 (6–10)	8 (3–10)
Cannabis resin (hashish)	9 (6–12)	10 (6–11)
Estimated purity (1–4 scale)		
Heroin	2 (1–2)	1 (1–2)
Cocaine	1.5 (1–2)	1 (1–2)
Cannabis	3 (2–4)	3 (2–3)
Impact of pandemic on substance use (1–5 scale, 1 = decreased, 3 = usual, 5 = increased)		
Heroin	3 (2–3)	2 (1–3)
Cocaine	3 (2–3)	2 (1–3)
Cannabis	3 (2–3)	3 (2–3)
Alcohol	3 (2–3)	3 (2–3)
Prescription drugs	3 (2.5–3)	3 (2–3)
Other illegal drug	3 (2.5–3)	1 (1–3)
Impact of pandemic on social situation and health (1–5 scale, 1 = no impact, 5 = a big impact)		
Social and financial situation	3 (1–3)	1 (1–3)
Fear of police controls	1 (1–3)	1 (1–3.5)
Stealing or racketeering of substances	1 (1–1)	1 (1–1)
Stress and anxiety	2 (1–4)	2 (1–3)
Mental health in general	2 (1–3)	2 (1–3)
Physical health in general	2 (1–3)	1 (1–3)

Notes: IQR = interquartile range (25th percentile–75th percentile); CHF = Swiss francs. Use patterns, purchases, prices, and estimated purity were evaluated only among participants having used the substance; for these questions, refer to first question about substance use for total *N*.

**Table 2:** Correlation between impact of pandemic on use of different substances.

	Heroin	Cocaine	Cannabis	Alcohol	Prescription Drugs	Other Drugs
Wave 1						
Heroin	1					
Cocaine	0.71 **	1				
Cannabis	0.51	0.66 **	1			
Alcohol	0.70 **	0.54 *	0.70 **	1		
Prescription drugs	0.42	0.00	−0.18	0.50	1	
Other drugs	0.00	−0.07	0.11	0.87 **	0.88 **	1
Wave 2						
Heroin	1					
Cocaine	0.70 **	1				
Cannabis	0.67 **	0.61 *	1			
Alcohol	0.75 **	0.59 *	0.65 *	1		
Prescription drugs	0.60 *	0.34	0.66 *	0.77 **	1	.
Other drugs	0.89 **	0.90 **	0.90 **	0.75 *	0.91 **	1

* = significant at the 0.05 level; ** = significant at the 0.01 level. Impact on substance use was measured on a 5-point Likert scale with anchors at 1 “decreased”, 3 “usual”, and 5 “increased”. Coefficients are Spearman’s rank correlations coefficients.

**Table 3:** Correlation between impact of pandemic on use of different substances and impact on health and social situation.

	Heroin	Cocaine	Cannabis	Alcohol	Prescription Drugs	Other Drugs
Wave 1						
Social and financial situation	0.02	–0.57 **	–0.59 **	–0.40	0.17	0.23
Fear of police controls	0.36	0.31	0.04	0.14	0.32	0.19
Stealing/racketeering of substances	0.15	–0.10	–0.18	0.06	0.16	0.14
Stress and anxiety	0.38	0.03	–0.06	0.37	0.59 **	0.50
Mental health in general	0.48 *	0.03	0.07	0.35	0.57 **	0.19
Physical health in general	0.17	–0.01	0.00	0.42	0.46 *	0.66
Wave 2						
Social and financial situation	–0.07	0.09	0.14	0.58 *	0.43	–0.08
Fear of police controls	0.17	0.17	0.30	0.40	0.28	0.00
Stealing/racketeering of substances	0.36	0.05	0.30	0.16	0.10	0.12
Stress and anxiety	0.31	0.25	0.38	0.66 **	0.51 *	0.54
Mental health in general	0.25	0.23	0.40	0.69 **	0.60 **	0.39
Physical health in general	–0.15	0.05	–0.01	0.49 *	0.45	0.01

* = significant at the 0.05 level; ** = significant at the 0.01 level. Impact on substance use was measured on a 5-point Likert scale with anchors at 1 “decreased”, 3 “usual”, and 5 “increased”. Impact on health and social situation was measured on a 5-point Likert scale with anchors at 1 “no impact”, and 5 “a big impact”. Coefficients are Spearman’s rank correlations coefficients.

## Data Availability

Data used in the current study are available from the corresponding author on reasonable request.

## Supplementary Materials

The following are available online at https://www.mdpi.com/article/10.3390/ijerph18094960/s1, File S1: Original quantitative questionnaire.
