# Supplementary material for: Evolution of the Illegal Substances Market and Substance Users’ Social Situation and Health during the COVID-19 Pandemic
Source: Int J Environ Res Public Health. 2021 May 7;18(9):4960. doi: 10.3390/ijerph18094960 (PMC8125553; doi:10.3390/ijerph18094960)
Supplement: Supplementary file 1 [file ijerph-18-04960-s001.zip › ijerph-1200179-supplementary.pdf]

Évaluation et suivi de l'évolution du marché des stupéfiants résultant de l'épidémie Covid-19

Questionnaire auprès des usagers de substances

Nous souhaitons comprendre l'évolution du marché des stupéfiants dans le contexte de l'épidémie du nouveau coronavirus pour mieux connaître la situation que rencontrent les consommateurs de stupéfiants et évaluer si les services actuels sont adaptés à leurs besoins.

Toutes les informations que vous nous donnez sont **totalement anonymes** et ne serviront qu'à comprendre la situation actuelle. Aucune donnée ne sera transmise plus loin.

Consommation de substances du marché noir

1. **Avez-vous consommé de l'héroïne au cours des 7 derniers jours ?** ❑ Non ❑ Oui

Si oui, à quelle fréquence ?

- Nombre de jours : ❑ 1 ❑ 2 ❑ 3 ❑ 4 ❑ 5 ❑ 6 ❑ 7
- Combien de fois par jour de consommation (en moyenne) : ______

Comment l'avez-vous obtenu ?
(Plusieurs réponses possibles)

- ❑ Stock personnel
- ❑ Reçu par un proche / ami-e
- ❑ Acheté à un consommateur
- ❑ Acheté à un trafiquant (quelqu'un qui ne consomme pas)

Sous quelle forme l'avez-vous acheté et à quel prix ?
(Plusieurs réponses possibles)

❑ En pacson / paquet	au prix de	____ Frs	pour	_____ g
❑ En grip / sachet	au prix de	____ Frs	pour	_____ g
❑ Autre, Préciser : ________________	au prix de	____ Frs	pour	_____ g

A votre avis, quelle est la pureté de l'héroïne que vous avez achetée ?

❑ Faible (0-10%)	❑ Moyenne (11-20%)	❑ Élevée (21-30%)	❑ Très élevée (+ de 30%)

2. **Avez-vous consommé de la cocaïne au cours des 7 derniers jours ?** ❑ Non ❑ Oui

Si oui, à quelle fréquence ?

- Nombre de jours : ❑ 1 ❑ 2 ❑ 3 ❑ 4 ❑ 5 ❑ 6 ❑ 7
- Combien de fois par jour de consommation (en moyenne) : ______

Comment l'avez-vous obtenu ?
(Plusieurs réponses possibles)

- ❑ Stock personnel
- ❑ Reçu par un proche / ami-e
- ❑ Acheté à un consommateur
- ❑ Acheté à un trafiquant (quelqu'un qui ne consomme pas)

Sous quelle forme l'avez-vous acheté et à quel prix ?
(Plusieurs réponses possibles)

❑ Petite boulette	au prix de	____ Frs	pour	_____ g
❑ Grosse boulette / parachute	au prix de	____ Frs	pour	_____ g
❑ Autre, Préciser : ________________	au prix de	____ Frs	pour	_____ g

A votre avis, quelle est la pureté de la cocaïne que vous avez achetée ?

❑ Faible (0-25%)	❑ Moyenne (26-50%)	❑ Élevée (51-75%)	❑ Très élevée (+ de 75%)

3. Avez-vous consommé du cannabis au cours des 7 derniers jours ? | ❑ **Non** | ❑ **Oui**

Si oui, à quelle fréquence ?

– Nombre de jours : ❑ 1 | ❑ 2 | ❑ 3 | ❑ 4 | ❑ 5 | ❑ 6 | ❑ 7

– Combien de fois par jour de consommation (en moyenne) : ______

Comment l'avez-vous obtenu ?
(Plusieurs réponses possibles)

- ❑ Stock personnel
- ❑ Reçu par un proche / ami-e
- ❑ Acheté à un consommateur
- ❑ Acheté à un trafiquant (quelqu'un qui ne consomme pas)

Sous quelle forme l'avez-vous acheté et à quel prix ?
(Plusieurs réponses possibles)

❑ Herbe	au prix de	_____ Frs	pour	______ g
❑ Résine (hash, shit)	au prix de	_____ Frs	pour	______ g
❑ Autre, Préciser : ________________	au prix de	_____ Frs	pour	______ g

A votre avis, quel est le taux de THC du cannabis que vous avez acheté ?

❑ Faible (0-5%)	❑ Moyen (6-10%)	❑ Élevé (11-15%)	❑ Très élevé (+ de 15%)

Impact de l'épidémie sur votre consommation et votre santé

La situation actuelle **d'épidémie** (disponibilité des drogues, distanciation sociale, etc.) a-t-elle une influence sur votre consommation :

	Diminuée		*Habituelle*		*Augmentée*	*Pas utilisé*
– d'héroïne	❑ 1	❑ 2	❑ 3	❑ 4	❑ 5	❑
– de cocaïne	❑ 1	❑ 2	❑ 3	❑ 4	❑ 5	❑
– de cannabis	❑ 1	❑ 2	❑ 3	❑ 4	❑ 5	❑
– d'alcool	❑ 1	❑ 2	❑ 3	❑ 4	❑ 5	❑
– de médicaments (par ex. benzo)	❑ 1	❑ 2	❑ 3	❑ 4	❑ 5	❑
– d'autres drogues	❑ 1	❑ 2	❑ 3	❑ 4	❑ 5	❑

La situation actuelle **d'épidémie** a-t-elle une influence sur votre situation et votre santé :

	Pas d'impact				*Un grand impact*
– situation sociale et financière	❑ 1	❑ 2	❑ 3	❑ 4	❑ 5
– peur des contrôles de police	❑ 1	❑ 2	❑ 3	❑ 4	❑ 5
– vol de produit, racket	❑ 1	❑ 2	❑ 3	❑ 4	❑ 5
– niveau de stress, d'angoisse	❑ 1	❑ 2	❑ 3	❑ 4	❑ 5
– santé mentale en générale	❑ 1	❑ 2	❑ 3	❑ 4	❑ 5
– santé physique en générale	❑ 1	❑ 2	❑ 3	❑ 4	❑ 5

A votre avis, comment pourrait-on mieux aider les consommateurs de stupéfiants dans la situation actuelle ?

Vous êtes…

Sexe : ❑ H | ❑ F   Age : ______   Situation professionnelle : ❑ Emploi | ❑ Autres

Merci beaucoup pour votre participation !
